# Synergistic Integration of Multimodal Metabolic and Bariatric Interventions Transforming Transplant Care

**DOI:** 10.3390/jcm14165669

**Published:** 2025-08-11

**Authors:** Donovan Hui, Alex C. Judd, Chioma Moneme, Heather Passerini, Stephanie Silpe, Alexander Podboy, Shawn J. Pelletier, Peter T. Hallowell, Thomas H. Shin

**Affiliations:** 1 Division of General Surgery, Department of Surgery, University of Virginia, Charlottesville, VA 22903, USA; 2 Division of Transplant Surgery, Department of Surgery, University of Virginia, Charlottesville, VA 22903, USA; 3 Division of Gastroenterology, Department of Medicine, University of Virginia, Charlottesville, VA 22903, USA

**Keywords:** bariatric surgery, transplant surgery, endobariatrics

## Abstract

Obesity presents a significant barrier to transplant eligibility due to increased morbidity associated with higher BMI. Patients with obesity who undergo transplantation face elevated risks of perioperative complications, morbidity from metabolic disease, and delayed graft function. However, recent advances in metabolic and bariatric medicine, endoscopy, and surgery offer promising opportunities for integration with transplant care. This critical review explores the potential benefits of metabolic and bariatric interventions for at-risk transplant patients. Here, we will briefly discuss the implications of obesity in transplant patients, pharmacologic, surgical, and endoscopic interventions, and ultimately, the role of bariatric surgery in different solid organ transplants. The successful implementation of these approaches could dramatically expand access to solid organ transplantation, creating life-saving opportunities for patients who would otherwise be deemed ineligible for this essential treatment. Despite the implications of metabolic and bariatric interventions in transplant care, this review is limited by the need for long-term studies of outcomes to better understand the effects of graft survival and durability of changes in metabolic syndromes.

## 1. Introduction

The global effect of morbid obesity has detrimental effects across all segments of patient populations, including patients requiring visceral transplant surgery. The prevalence of adult obesity in the United States, as estimated by the Centers for Disease Control and Prevention (CDC) in 2023, is 40.3%. Among individuals eligible for solid organ transplantation, the prevalence of obesity is even higher, reaching upwards of 46% [1,2]. This high prevalence significantly impacts transplant outcomes, leading to increased risks of graft failure, prolonged hospitalization, impaired healing, rejection, and mortality [3,4,5]. Consequently, many centers consider obesity a relative contraindication, particularly for candidates with a body mass index (BMI) greater than 40 kg/m^2^.

The severity of obesity is determined by BMI and stratified by class (class 1: 30–34.9 kg/m^2^, class 2: 35–39.9 kg/m^2^, class 3: ≥40 kg/m^2^) [6]. Current eligibility criteria for metabolic and bariatric surgery (MBS) are BMI > 40 kg/m^2^ or BMI > 35 kg/m^2^ with metabolic disease. Recent international consensus has reconsidered these criteria and would recommend metabolic and bariatric surgery for a BMI > 35 kg/m^2^, regardless of the presence of comorbidities or a BMI of 30–35 kg/m^2^ with metabolic disease [7]. However, BMI has limitations in transplant populations, particularly in end-stage organ dysfunction with sarcopenia, anasarca, and ascites in the setting of obesity [8]. Imaging studies such as dual-energy X-ray absorptiometry (DEXA), bioelectrical impedance assessment (BIA), and computer tomography (CT), as well as functional, muscle strength, inflammatory, and metabolic biomarkers have been studied to assess sarcopenia. Although there is no standardization of biomarkers and adjustments in BMI for ascites have shown no predictive changes in outcomes, sarcopenia in and of itself is associated with worse outcomes with longer hospital stays and worse postoperative morbidity [8,9,10,11]. Integrated metabolic and bariatric centers can help differentiate changes in muscle mass from visceral fat loss, improving metabolic health assessment [12].

While MBS effectively manages long-term obesity, its peritransplant role remains underexplored. This review examines evidence for bariatric surgery and other weight management strategies in transplant care, investigating how integrated approaches can optimize outcomes across solid organ transplantation.

## 2. The Impact of Obesity

Although not all end-organ dysfunction is a product of obesity, it accelerates multiple pathways to end-stage organ dysfunction, including diabetes, hypertension, cardiovascular disease, and nonalcoholic fatty liver disease (NFLD), now termed metabolic dysfunction-associated liver disease (MASLD) [13,14]. These conditions increase surgical risks, particularly wound infections, thromboembolism, renal failure, and prolonged operative times [15,16,17]. The presence of obesity in already high-risk patients poses significant considerations that can increase time on the transplant waitlist and subsequently decrease the likelihood of a timely transplant. Despite being a major contributor to organ failure, severe obesity remains a relative contraindication for transplantation, with few centers accepting candidates with a BMI > 40 kg/m^2^ [18]. This restriction stems from documented perioperative complications, including increased operative times, bleeding, infections, wound dehiscence, biliary, and metabolic complications. Post-transplant weight gain presents additional challenges, though metabolic and bariatric surgery can mitigate these risks by reducing weight gain and associated metabolic complications [19,20].

## 3. Pharmacologic Weight Loss in Transplant Patients

While various options exist for weight loss, it is essential to consider patient adherence, comorbid conditions, and insurance/financial impact on method selection. While lifestyle modifications are available for weight loss, they are less effective and surprisingly more costly long-term than medications or surgery [21,22]. GLP-1 receptor agonists (GLP1RAs), including liraglutide, semaglutide, and tirzepatide, have emerged as leading pharmacotherapy options, demonstrating superior weight loss effects compared to previous generations of anti-obesity medications and appetite suppressants [23,24,25].

Though these medications are both safe and efficacious for pre-transplant candidates, access may be limited, and all are associated with significant rates of gastrointestinal adverse reactions, including nausea, vomiting, and gastroparesis [24,26]. Other GI complications may include cholelithiasis or cholecystitis (<3.5%) or pancreatitis (<2%), with the use of GLP1RAs [24,27]. While the incidence of cholelithiasis and cholecystitis is yet undefined, it is known that gallstone formation following bariatric surgery has an incidence of 10–38%, and treatment with ursodeoxycholic acid (UDCA) may be warranted in GLP1RA use [27].

Although the data is sparse, evidence supports GLP1RA safety and efficacy across all solid transplant patients with severe obesity without significant effects on immunosuppression or post-transplant graft function [22,23,28,29,30,31]. In a study by Dotan et al., GLP1RAs significantly reduced the incidence of major cardiovascular events (MACE) and all-cause mortality in solid organ transplant recipients with diabetes, with a hazard ratio (HR) of 0.46 for MACE and 0.39 for all-cause mortality compared to non-users [32]. However, further research is needed regarding optimal dosing and effects on sarcopenia in transplant populations with larger prospective trials.

## 4. Synergistic Integration of Metabolic and Bariatric Surgery in Solid Organ Transplant Care

MBS offers comprehensive benefits, reducing antihypertensive and insulin requirements while potentially altering the trajectory of end-stage organ dysfunction [19]. Weight loss can be particularly difficult for patients with end-stage organ disease. Physiological reserve and exercise tolerance in transplant patients can be low, limiting the ability of high-risk obese transplant patients to lose weight. Retrospective studies have demonstrated that patients with obesity and chronic kidney disease requiring dialysis have limited pharmacologic options with a high risk of weight regain following cessation of medical therapies [33,34].

Surgical interventions can alter the trajectory of end-stage organ dysfunction by reducing multiple obesity-related risks and potentially bridge patients to improved survival following transplantation [5].

Current guidelines recommend metabolic and bariatric surgery prior to transplantation when possible, with at least 1-year post-transplant stability required for those requiring surgery after transplant [19]. MBS before transplantation is preferred, particularly as pre-transplant weight loss can improve metabolic syndrome, inflammation, hypertension, and diabetes. Significant regulated weight loss can allow for transition into transplant eligibility and even improve chronic disease states for which transplant is no longer warranted [19,35]. By mitigating underlying health conditions, MBS can not only enhance transplant eligibility but also improve long-term survival rates by improving organ function and reducing complications. Further integration between MBS and transplant evaluation is an area of significant need for further research and development to decrease morbidity and mortality for pre-transplant patients [4,36].

## 5. Endobariatrics in Transplant

Endoscopic bariatric therapies (EBTs) describe minimally invasive endoscopic treatments designed to manage obesity and its associated conditions. Multiple EBTs continue to be developed, including intragastric balloons, aspiration therapy, endoscopic sleeve gastroplasty, as well as small bowel devices and remodeling techniques [37,38]. While surgery remains the gold standard in the management of obesity, endobariatrics offer less invasive alternatives with potentially lower periprocedural complications and faster recovery times [39].

The American Gastroenterological Association (AGA) recommends considering EBTs in patients with severe obesity as a bridge to traditional bariatric surgery or other interventions, including organ transplantation, when weight limits are a barrier [40]. The two most commonly evaluated endobariatric therapies for the treatment of transplant-associated obesity are intragastric balloon therapy and endoscopic sleeve gastroplasty (ESG).

Intragastric balloon (IGB) therapy utilizes an endoscopically removable space-occupying balloon to promote early satiety, delayed gastric emptying, and limit caloric intake. IGB therapy has been explored in patients awaiting kidney, heart, and liver transplantation, proving safe but with potential adverse outcomes such as significant nausea, balloon migration, and esophageal tears [41,42,43,44]. Endoscopic sleeve gastroplasty reduces gastric volume through placement of full-thickness sutures to create a sleeve-like structure [39]. EBTs have shown significant improvements in hepatic dysfunction in MASLD and reversal of fibrosis [45]. However, ESG is contraindicated in end-stage liver disease due to increased portal pressures and bleeding risks [46].

Endobariatrics remains a relatively new approach in transplant care and can be a potential intervention to improve both pre- and post-transplant outcomes, especially in patients with contraindications to surgery. Given the current limitations and short-term follow-up on patients undergoing EBT, further research is needed to establish the role and long-term efficacy of endobariatrics in transplant populations.

## 6. Metabolic and Bariatric Surgery in Solid Organ Transplant

The published literature on this bariatric surgery population primarily consists of retrospective analyses from institutional case series or database studies, which feature a limited patient population and lack reports on long-term transplant outcomes. 

### 6.1. Kidney Transplant

Diabetes and hypertension drive end-stage renal disease (ESRD) development, with obesity accelerating progression through multiple metabolic pathways [47,48,49]. The prevalence of obesity in ESRD reaches 39% [50]. The BMI cutoff for entering a renal transplant program varies by center, with the majority excluding patients who have a BMI > 35 kg/m^2^ [51]. The 2022 OPTN/SPTR database shows 140,165 candidates listed for kidney transplant, with 26,306 completing transplantation. Notably, 46.3% of listed candidates had obesity (27.4% BMI 30–34.9 kg/m^2^ and 18.9% BMI > 35 kg/m^2^) [2].

Despite the obesity paradox conferring survival advantage in ESRD patients on dialysis, kidney transplantation provides significant survival benefit even among obese patients, though with decreased advantage for BMI > 40 kg/m^2^ (risk reduction 48% versus 66% in other BMI groups) [52,53,54]. Multivariable analyses show that obesity alone does not independently predict decreased graft survival or rejection, though it is associated with other independent predictors like diabetes, hypertension, and expanded donor criteria [55,56,57,58,59,60,61,62,63]. A single-center analysis of transplants with a BMI ≥ 40 kg/m^2^ showed longer dialysis duration and lower KDPI kidneys, but no significant differences in delayed graft function, reoperations, readmissions, wound complications, survival, or renal function at 1 year [64].

Metabolic and bariatric surgery offers significant benefits, with meta-analyses showing 50.3% of ESRD patients achieve transplant-eligible weight, and 29.5% receive transplantation [4,65]. However, ESRD patients experience longer hospital stays, higher morbidity, and increased risk of dehydration affecting renal function. Sleeve gastrectomy is preferred over Roux-en-Y bypass, with no significant immunosuppression dose adjustments needed [66,67,68]. Post-transplant bariatric surgery carries additional complications, though weight loss outcomes match non-ESRD populations [69]. However, long-term outcome data is limited in this patient population. Nonetheless, limited data shows improved graft survival in the perioperative period while having comparable graft survival to non-obese patients at up to 5 years of follow-up [70,71,72].

### 6.2. Liver Transplant

MASLD affects up to 30% globally and 68% of obese patients in the United States [73]. MASLD with progression to metabolic dysfunction-associated steatohepatitis (MASH) can lead to cirrhosis and hepatocellular carcinoma, requiring transplantation [74]. MASH is projected to become the leading transplant indication [75]. Metabolic surgery can resolve hepatic steatosis and fibrosis in 66% and 40–50% of patients, respectively, though it is contraindicated in decompensated cirrhosis due to mortality risks [8,74,76].

The 2022 OPTN/SRTR data shows 24,186 adult candidates listed for liver transplant, with 9001 undergoing transplantation. Obesity was present in 41.3% of MASH candidates [77]. While most centers limit BMI to 35–40 kg/m^2^, patients with BMI > 40 kg/m^2^ still show overall survival benefit after transplant [78]. The obesity paradox exists in stable cirrhosis, but obese transplant candidates have higher waitlist morbidity and Model for End-Stage Liver Disease (MELD) scores [8]. Although obesity’s impact on long-term outcomes remains controversial, it increases operative times, blood loss, length of stay, and infections [3,33,59,79,80].

Bariatric surgery outperforms medical treatment alone [81]. For pretransplant candidates, eligibility requires MELD < 15 with minimal cirrhosis, varices, and encephalopathy, without significant coagulopathy or malnutrition [19]. Sleeve gastrectomy is preferred for its lower risk and preserved biliary access [19,82]. Patients achieve a mean 50% excess body weight loss without procedure-associated mortality [83]. Meta-analysis shows that 58.5% of candidates achieve transplant-eligible weight, with 21.9% receiving transplants [4]. Data on the timing of bariatric surgery before, during, or after liver transplant is limited, but meta-analysis suggests that there is no significant difference in bariatric complications such as bleeding and staple line leak rates. Further 3-year follow-up shows simultaneous surgery having more durable post-transplant weight loss and control of metabolic syndromes [8,84,85,86]. Although long-term data beyond 5 years on the benefits of bariatric surgery in liver transplant patients is limited, a combined liver transplant with sleeve gastrectomy may offer improvement in diabetes, hypertension, and hyperlipidemia [85]. Post-transplant bariatric surgery requires one-year stability but carries increased operative times and morbidity associated with increased complexity following transplant surgery [87].

### 6.3. Pancreas Transplant

Type 1 diabetes remains the main reason for requiring a pancreas transplant. The rate of obesity in Type 1 diabetic patients, while historically low, has increased in recent years, mirroring the trend of the general population [88]. Data from the 2022 OPTN/SRTR show that rates of pancreas transplantation for Type 2 diabetes have tripled over the past decade, now accounting for over 20% of all transplants [89]. As a result, and as seen with other solid organs, the proportion of obese patients on the waitlist for pancreas transplantation has steadily increased [83]. Selection criteria for pre-transplant bariatric surgery are particularly important for pancreas recipients as many patients have other end-organ dysfunction from long-standing diabetes, including gastroparesis and renal failure [90]. The inclusion criteria have expanded to allow for pancreas transplantation, most commonly simultaneous pancreas and kidney transplantation (SPK) or pancreas after kidney transplantation (PAK), for many patients with Type 2 Diabetes, as concomitant ESRD is associated with worse outcomes [91]. A recent review article found that patients with Type 2 Diabetes who receive pancreas transplantation have higher average BMIs and are more likely to have a BMI > 28 than those with Type 1 Diabetes, yet outcomes remain comparable [88].

Historically, pancreas transplantation excluded obese patients with a BMI > 30 kg/m^2^ due to higher rates of wound complications, infection, rejection, graft loss, and death [91,92]. While some studies show similar graft loss and mortality between obese and non-obese patients, obesity increases rejection risk [93,94]. Despite these risks, 23% of listed pancreas transplant candidates now have a BMI > 30 kg/m^2^ [89].

Robotic pancreas transplantation decreases infectious and wound complications, offering safer and expanded access to transplant candidates with obesity. A recent case-control study demonstrates that bariatric surgery prior to pancreas transplantation is feasible and safe, and expands access for patients with prohibitive obesity. Sleeve gastrectomy appeared to be the preferred surgical choice due to malabsorptive and anatomical concerns. Nevertheless, gastric bypass remains a viable option, particularly favoring allograft anastomosis located distal to the jejuno-jejunal anastomosis. Patients were able to maintain weight loss and reached an acceptable BMI threshold for transplantation, with superior 4-year graft and patient survival rates of 100% [95].

### 6.4. Heart Transplant

Obesity significantly impacts heart failure risk, increasing by 5–7% for each BMI point [96]. Higher BMI correlates with worse post-transplant outcomes, showing a stepwise increase in death or re-transplantation risk [97,98]. BMI > 40 kg/m^2^ specifically shows an increased hazard ratio for waitlist death, longer wait times, and the highest post-transplant mortality [5]. Outcomes vary for BMI 30–35 kg/m^2^, showing increased bypass time and early complications but no long-term survival differences [99,100]. As a result, class 2 obesity and above is considered a relative contraindication to active listing. The 2022 OPTN/SRTR data reveals 7519 adult cardiac transplant candidates with 3668 patients undergoing transplants, of which 14% are in combination with another solid organ [101]. Historical data from 2006 to 2020 showed that 32.8% of listed patients had a BMI > 30 kg/m^2^, with significant correlations between increasing BMI and longer cardiac transplant wait times and left ventricular assist device (LVAD) utilization [5].

Bariatric surgery effectively counters obesity’s cardiac impact, with Swedish Registry data showing a five-fold lower heart failure risk post-surgery [102]. Propensity-matched cohort studies demonstrate prior bariatric surgery was associated with a 50% reduction in mortality and a shorter length of stay during heart failure admissions, while other comparative retrospective data reveals MBS leading to improved left ventricular ejection fraction (LVEF) and New York Heart Association (NYHA) class [103,104]. The cardioprotective effects of MBS have been successfully leveraged as a bridge to heart transplantation in conjunction with LVADs for transplant candidates with severe obesity in order to achieve transplant waitlist criteria [105,106].

Meta-analyses of heart failure patients with BMI > 35 kg/m^2^ report extremely low 30-day mortality rates after bariatric surgery, with 40–57% of patients achieving transplantation and 8.5% of post-MBS patients showing sufficient LVEF improvement to avoid transplant in addition to decreased need for long-term post-transplant diuretic and vasodilator requirements [19,107].

Metabolic and bariatric surgery in patients with heart failure has a higher risk of morbidity and longer hospital stays, but is still considered safe [108]. Simultaneous metabolic and cardiac transplant has also been assessed in a more limited meta-analysis showing no significant long-term differences in outcomes compared to staged surgeries, but carries a mortality rate of 15%. While this is considerably higher than MBS in non-heart failure patients, it is lower than the one-year mortality risk of LVAD patients with severe obesity managed non-surgically [19,109].

### 6.5. Lung Transplant

Obesity significantly impacts pulmonary function through mechanical restriction and inflammatory adaptations, reducing functional residual capacity by up to 33% in severe obesity [110,111]. Obesity in and of itself may not play an important role in ventilatory outcomes, with only 15% of patients with obesity having restrictive lung disease, but it can greatly contribute to the restrictive processes affecting lung function by affecting thoracic expansion [112,113]. The 2022 OPTN/SRTR data shows that 4228 candidates were listed for lung transplant, of which 2031 patients had restrictive lung disease, and 2743 patients underwent lung transplantation [114].

Obesity in lung transplantation has had varying outcomes with mixed reports regarding its safety profile. Multiple studies identify severe obesity as an independent risk factor for primary graft dysfunction and morbidity within the first year post-lung transplant, particularly affecting 90-day and 1-year mortality for lung transplantations for restrictive lung disease [115,116]. Retrospective analysis of lung transplant recipients with severe obesity reveals that BMI > 30 kg/m^2^ at the time of transplant is associated with greater length of mechanical ventilation, hospital length of stay, and composite post-transplant complications, regardless of starting BMI at the time of waitlist [117].

Robust clinical evidence describing outcomes of lung transplantation in patients with severe obesity remains limited. Current consensus guidelines categorize lung transplant recipients with a BMI > 30 kg/m^2^ as high-risk, and limited select specialized centers accept transplant candidates with a BMI > 35 kg/m^2^ [118,119]. Despite the physiologic complications associated with end-stage lung disease, such as pulmonary fibrosis and pulmonary hypertension, various case series demonstrate the safety of MBS within this patient population, with the recommendation for Roux-en-Y gastric bypass as MBS of choice given the significant role of gastroesophageal reflux disease in interstitial lung disease exacerbation. Furthermore, improvement in pulmonary function tests following MBS suggests a reduction in pulmonary complications at the time of lung transplant [120,121,122].

## 7. Conclusions

Bariatric and metabolic surgery has transformed transplant care for patients previously excluded due to high BMIs. While obesity can worsen both pre- and post-transplant outcomes, including increased surgical complications and mortality risks, metabolic and bariatric interventions offer solutions beyond weight loss alone. These interventions can improve comorbidities like hypertension, diabetes, and cardiovascular disease, sometimes eliminating the need for transplantation entirely. The emerging integration of pharmacologic agents and endobariatrics with metabolic surgery provides new opportunities for sustainable weight management. This multifaceted approach promises to revolutionize how transplant centers manage obese candidates. Further studies in the long-term outcomes of bariatric surgery in solid organ transplant are needed to better understand differences in long-term survival, quality of life, primary and secondary graft survival, and durability of changes in metabolic syndromes (Table 1).

## Figures and Tables

**Table 1:** Summary of the literature for bariatric surgery in relation to solid organ transplant type.

Organ	Recommended BMI	Perioperative Considerations	Preferred MBS	Notes
Kidney	BMI > 40 kg/m^2^	Dehydration worsening, renal failure	Sleeve	Obesity paradox with no survival benefits in BMI > 40 kg/m^2^
Liver	BMI 35–40 kg/m^2^	Risk of hepatic decompensation. MBS after liver transplant with longer operative times and complications with associated adhesions	Simultaneous liver transplant and sleeve	MBS is effective if MELD < 15 and no decompensated cirrhosis; sleeve preferred
Pancreas	BMI > 30 kg/m^2^	Careful glycemic control with change in diet	Sleeve	Limited data, but may decrease transplant rejection.
Heart	BMI > 35 kg/m^2^	Adjustment to heart failure medications. High risk of mortality	No preference	MBS improves listing eligibility; often combined with LVAD bridging.
Lung	BMI > 30 kg/m^2^ (BMI > 35 for high volume centers)	Higher risk of pulmonary complications with prolonged mechanical ventilation	Gastric bypass	Higher risk of aspiration pneumonia with sleeve gastrectomy

## Abbreviations

The following abbreviations are used in this manuscript:

CDC	Centers for Disease Control and Prevention
BMI	Body mass index
DEXA	Dual-energy X-ray absorptiometry
BIA	Bioelectrical impedance assessment
CT	Computer tomography
MBS	Metabolic and bariatric surgery
NFLD	Nonalcoholic fatty liver disease
MASLD	Metabolic dysfunction-associated liver disease
GLP1RA	GLP-1 receptor agonist
UDCA	Ursodeoxycholic acid
MACE	Major cardiovascular event
HR	Hazard ratio
EBT	Endoscopic bariatric therapy
AGA	American Gastroenterological Association
ESG	Endoscopic sleeve gastrectomy
IGB	Intragastric balloon
ESRD	End-stage renal disease
OPTN	Organ Procurement and Transplantation Network
SRTR	Scientific Registry of Transplant Recipients
KDPI	Kidney donor profile index
MASH	Metabolic dysfunction-associated steatohepatitis
MELD	Model for End-Stage Liver Disease
PAK	Pancreas after kidney transplantation
SPK	Simultaneous pancreas and kidney transplantation
LVAD	Left ventricular assist device
LVEF	Left ventricular ejection fraction
NYHA	New York Heart Association
